# Rojiroti microfinance and child nutrition: a cluster randomised trial

**DOI:** 10.1136/archdischild-2018-316471

**Published:** 2019-10-10

**Authors:** Shalini Ojha, Lisa Szatkowski, Ranjeet Sinha, Gil Yaron, Andrew Fogarty, Stephen John Allen, Sunil Choudhary, Alan Robert Smyth

**Affiliations:** 1 Division of Medical Sciences and Graduate Entry Medicine, University of Nottingham, Nottingham, UK; 2 Division of Epidemiology & Public Health, University of Nottingham, Nottingham, UK; 3 Department of Community Medicine, Patna Medical College, Patna, Bihar, India; 4 GY Associates Ltd and Trustee, Harpenden, UK; 5 Department of Child Health, The Liverpool School of Tropical Medicine, Liverpool, UK; 6 Centre for Promoting Sustainable Livelihood (CPSL), Patna, Bihar, India; 7 Division of Child Health, Obstetrics & Gynaecology, Nottingham University, Nottingham, UK

**Keywords:** microfinance, low & middle income countries, wasting, child growth, weight for height z score

## Abstract

**Objective:**

To determine whether Rojiroti microfinance, for poor Indian women, improves child nutrition.

**Design:**

Cluster randomised trial.

**Setting:**

Tolas (village communities) in Bihar State.

**Participants:**

Women and children under 5 years.

**Interventions:**

With Rojiroti microfinance, women form self-help groups and save their money to provide loans to group members. After 6 months, they receive larger external loans. Tolas were randomised to receive Rojiroti immediately or after 18 months.

**Outcome measures:**

The primary analysis compared the mean weight for height Z score (WHZ) of children under 5 years in the intervention versus control tolas who attended for weight and height measurement 18 months after randomisation. Secondary outcomes were weight for age Z score (WAZ), height for age Z score, mid-upper arm circumference (MUAC), wasting, underweight and stunting.

**Results:**

We randomised 28 tolas to each arm and collected data from 2469 children (1560 mothers) at baseline and 2064 children (1326 mothers) at follow-up. WHZ was calculated for 1718 children at baseline and 1377 (674 intervention and 703 control) at follow-up. At 18 months, mean WHZ was significantly higher for intervention (−1.02) versus controls (−1.37; regression coefficient adjusted for clustering β**=**0.38, 95% CI 0.16 to 0.61, p=0.001). Significantly fewer children were wasted in the intervention group (122, 18%) versus control (200, 29%; OR=0.46, 95% CI 0.28 to 0.74, p=0.002). Mean WAZ was better in the intervention group (−2.13 vs −2.37; β**=**0.27, 95% CI 0.11 to 0.43, p=0.001) as was MUAC (13.6 cm vs 13.4 cm; β**=**0.22, 95% CI 0.03 to 0.40, p=0.02). In an analysis adjusting for baseline nutritional measures (259 intervention children and 300 control), only WAZ and % underweight showed significant differences in favour of the intervention.

**Conclusion:**

In marginalised communities in rural India, child nutrition was better in those who received Rojiroti microfinance, compared with controls.

**Trial registration number:**

NCT01845545.

**What is already known on this topic?**

- Microfinance programmes have been implemented widely in poor communities in low-income and middle-income countries.
- Some microfinance programmes have brought economic benefits to female participants.
- Studies evaluating the impact of microfinance on child health and nutrition have not been rigorous, and results have been conflicting.

**What this study adds?**

- In a cluster randomised trial, we found that several indices of child nutrition were better, at 18 months, in the groups randomised to Rojiroti microfinance.
- Weight for height Z score (primary outcome) was significantly better in the intervention group (−1.02) versus controls (−1.37).
- In poor and marginalised communities in Bihar, Rojiroti microfinance appears to prevent a deterioration in nutritional indices, in children under five, at times of food insecurity.

## Introduction

Globally, 50 million children under 5 years suffer from acute malnutrition or wasting (weight for height Z score (WHZ) of below −2).^1^ These children are at least three times more likely to die than their better nourished peers.^2^ Two-thirds live in Asia.^1^ Ending all forms of child malnutrition by 2030 is a Sustainable Development Goal.^3^ In spite of programmes to address malnutrition in almost all low-income and middle-income countries, acute malnutrition remains highly prevalent.^4^

Child health follows a social gradient where wealthier means healthier.^5^ Recent economic growth in India has not led to a reduction in childhood undernutrition.^6^ Bihar (population 116 million) is one of the poorest and most deprived states in India (population 1.3 billion). Nearly 90% of the population is rural and has poor access to healthcare and education. Of the 100 districts in India with the highest prevalence of malnutrition, 23 are in Bihar.^7^ Rojiroti (‘daily bread’) microfinance has been operating in Bihar since 2001.^8^ Participants are women, and 62% are from scheduled castes (disadvantaged groups, recognised in the Indian Constitution).^8^ It is delivered by the non-governmental organisation the Centre for Promoting Sustainable Livelihood (CPSL) (https://www.rojiroti.org). Women form self-help groups (SHGs) and contribute their own savings to a fund, from which they can request small loans. Later, women may become eligible for larger loans funded by CPSL (see box 1).^9^

Box 1 **The principles of Rojiroti microfinance**

- Women are asked to form self-help groups (SHGs).
- They contribute small amounts of money to a communal fund (approximately Rs2.5, or US 4 cents, per member per week).
- Women are expected to attend at least four SHG meetings (held weekly), before their savings entitle them to a loan.
- These loans are initially small (70 cents) and come from the SHG fund.
- After 6 months, women in the SHG are entitled to receive larger, external loans from CPSL of between Rs500 ($7) and Rs3 000 ($44), provided SHG credit is good.
- There are no restrictions on the use of loans.

Our hypothesis was that Rojiroti microfinance would improve nutrition among children under 5 years. We tested this through a cluster randomised trial, based in rural tolas (village neighbourhoods of around 500 people of similar socioeconomic status and caste).

## Methods

### Study design

We conducted a matched pair, cluster randomised controlled trial (RCT) with a 1:1 allocation ratio. The protocol has been published,^9^ and the trial is registered. Our findings are reported in line with the Consolidated Standards of Reporting Trials (CONSORT) extension for cluster randomised trials.^10^

**Figure 1:**
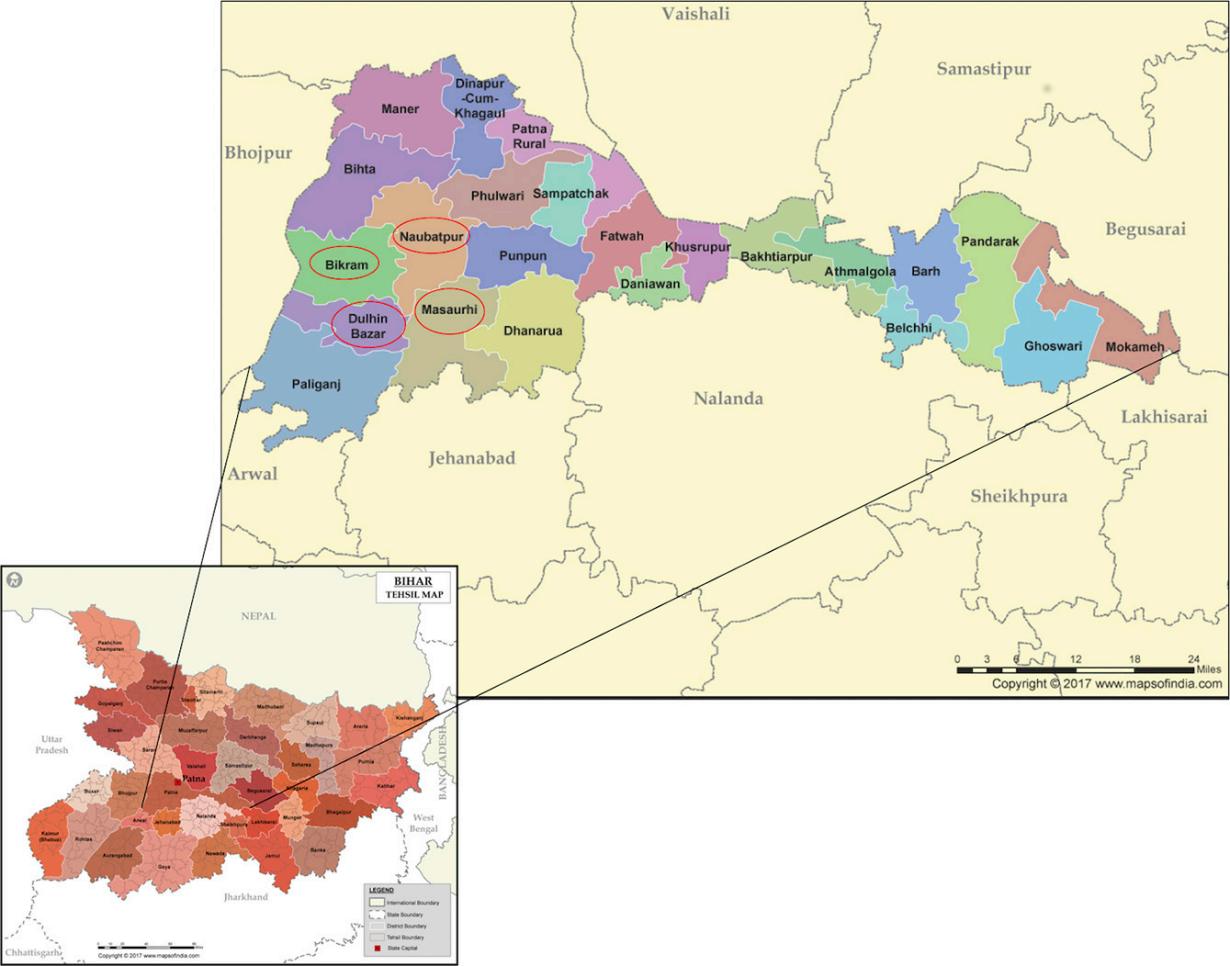
The state of Bihar, showing Patna district, with the participating administrative blocks (‘tehsils’) of Dulhin Bazar, Naubatpur, Masurhi and Bikram.

Participants were village women, mostly from scheduled castes. Four administrative blocks in Patna District (Dulhin Bazar, Naubatpur, Masaurhi and Bikram; figure 1) were chosen because of proximity to the teams from CPSL and Patna Medical College. We approached the next 60 tolas, due to be offered Rojiroti (on the basis of need). SHG membership was open to any woman in the tola. All children in the tola were invited for weighing and measuring, irrespective of whether their mother was an SHG member. There were no exclusion criteria.

### Consent and randomisation

We followed the CONSORT guidelines on consent for cluster randomised trials.^11^ Only 46% of women in rural Bihar are literate^12^ and so trial information was conveyed verbally (in Hindi). A CPSL volunteer explained that:

- The trial involved random allocation to immediate or delayed Rojiroti.
- In the delayed (control) group, participants could not implement Rojiroti for 18 months.
- Tolas declining the trial could access Rojiroti through the normal procedure.
- In control tolas, women could join non-Rojiroti SHGs.

Women indicated if they agreed or declined to participate by show of hands. The discussion and vote were video recorded. Written informed consent was obtained from one literate representative of the women. Consent for the child to participate was confirmed verbally when the children were weighed and measured.

Following consent, tolas of similar size were paired by researchers in Patna and given a tola ID by the research team in Nottingham. Tolas in each pair were randomly assigned to receive Rojiroti immediately (intervention) or after the final 18 month measurements (control) by the blinded researchers in Nottingham, using a computer-generated random number table. Intervention tolas were at least 15 km from any control tola to minimise ‘viral spread’ of the intervention. CPSL then implemented Rojiroti in the intervention tolas, starting with establishing SHGs. Data analysts, but not field workers, were blinded to allocation. The tola was the unit of randomisation and the child the unit of analysis.

### Procedures

Phase 1 (feasibility) recruitment took place August–September 2013 (20 tolas). Once feasibility was established, the pilot stage commenced, with phase 2 recruitment (30 tolas February–March 2014). Recruitment stopped during the monsoon, and phase 3 recruitment (six tolas) was in September 2014. At baseline, we collected data about each tola and demographic data for each mother and child. The age, sex, weight, length/height and mid-upper arm circumference (MUAC) were recorded for all children under 5 years of age (and over 6 months for MUAC).^9^ We used the following equipment for the age groups listed.

- Length: (<2 years) Seca 210 Measuring Mat. Standing height: (≥2 years) Seca 213 Portable Stadiometer (both Seca, Birmingham, UK).
- Weight: (<6 months) Docbel Baby Scales (Popular, Docbel Industries, New Delhi, India); (6 months–2 years) hanging scales (Venus CHS, Ace, Rajasthan, India); (≥2 years) Libra Fitness Standing Scale (Edryl, Goa, India).
- MUAC: (>6 months) MUAC tapes (Unicef Supply Division).

Equipment was calibrated, using standard measures, at the beginning of each visit. After setting the scales to zero, each child was weighed and measured three times, and the middle value used.^13^ Children were weighed and measured by CPSL workers, who were trained by staff from Patna Medical College, prior to the baseline and 18 month visits. Rojiroti then began in the tolas randomised to the intervention. CPSL staff met with women regularly to record how loans were used.

At 18 months, all children under five present in the tolas were invited for weighing and measurement. Mothers and children who were also present at the baseline survey were identified by unique ID codes. CPSL staff conducted one visit to each tola (to weigh and measure the children) at baseline and follow-up. Data were recorded on paper forms, entered electronically in Patna and transferred to Nottingham for analysis. In Nottingham, a random 10% of the electronic data were checked against the paper records. Errors were found in <1% of those checked.

### Statistical analysis

Data were checked for spurious age, height and weight entries using the Emergency Nutrition Assessment (ENA) Tool and implausible data excluded.^14^ Analysis was performed using Stata V.14.

#### Outcome measures

In our feasibility phase, the primary outcome was mortality, and this was registered in ClinicalTrials.gov in April 2013. The feasibility phase began in August 2013, but it proved impossible to collect reliable data on mortality. The primary outcome was therefore changed to mean WHZ). This is recorded in our published protocol^9^ in July 2014 and subsequently amended on ClinicalTrials.gov. Data collection was completed in March 2016.

We assessed the following primary and secondary outcomes at 18 months.

#### Primary outcome

**Primary outcome:** mean WHZ.

### Secondary outcomes

- Mean weight for age Z score (WAZ) and height for age Z score (HAZ).
- Prevalence of moderate to severe: wasting (WHZ below −2SD), undernutrition (WAZ below −2SD) and stunting (HAZ below −2SD).
- Mean MUAC.
- Prevalence of: moderate acute malnutrition (MUAC 12.5–11.5 cm) and severe malnutrition (MUAC <11.5 cm).

WHZ, HAZ and WAZ were calculated from the 2006 WHO growth standards,^15^ using the ‘zscore06’ function in Stata.

#### Sample size calculation

We used data from the feasibility phase on WHZ and the number of children per tola to determine the sample size and the number of tolas needed.^9^ Previous work has suggested that, where programmes achieve an improvement in Z score of 0.1 to 0.5, this has a meaningful effect on the prevalence of malnutrition in young children.^16^ There were on average 40 children under five in each of the first 20 tolas and the mean baseline WHZ score was −0.96 (SD 1.04). Baseline intracluster correlation coefficient was 0.082. We used the Stata function ‘clustersampsi’, which implements the sample size calculation procedures detailed by Hayes and Bennett.^17^ We calculated that, recruiting 60 tolas and allowing for 10% attrition, gave 80% power (alpha 0.05) to detect a difference in mean WHZ score of 0.26 SD between groups. We therefore decided to continue the trial, with the aim of recruiting 60 tolas, as there would be sufficient statistical power to detect a meaningful difference (if present) in the primary outcome.

In cross-sectional analyses, mean (SD) WHZ, HAZ, WAZ, MUAC and the prevalence of binary outcomes were calculated at 18 months. Linear or logistic regression models were used to quantify the difference in outcomes between children in intervention and control tolas at follow-up, using a multilevel model, with tola ID as a random effects variable, to account for clustering by tola. We intended to adjust for baseline nutritional status. However, Bihar has a transient population, with some families moving away and others arriving in the tola during the trial. Many of the children present at baseline did not attend follow-up, and additional children were measured at follow-up who were not present at baseline. We therefore conducted an analysis of children weighed and measured at follow-up without adjusting for baseline values and a second analysis of those present both at baseline and follow-up, adjusting for baseline nutritional status. We included in the model, a priori, the variables age, sex and number of children <5 in the family. No other variables were significant in the model.

We measured two potential harms of the programme in participating women at 18 months, using the χ^2^ test.

- Freedom to travel without the permission of a male relative—usually the husband (travel might be restricted if Rojiroti participation causes domestic disputes).
- Forced asset sales (which might arise if Rojiroti increased indebtedness).

We performed a post hoc analysis that was not specified in our protocol (results in online supplementary material). This was to determine whether the effects of the intervention varied according to mothers’ SHG membership.

10.1136/archdischild-2018-316471.supp1 Supplementary data

## Results

**Figure 2:**
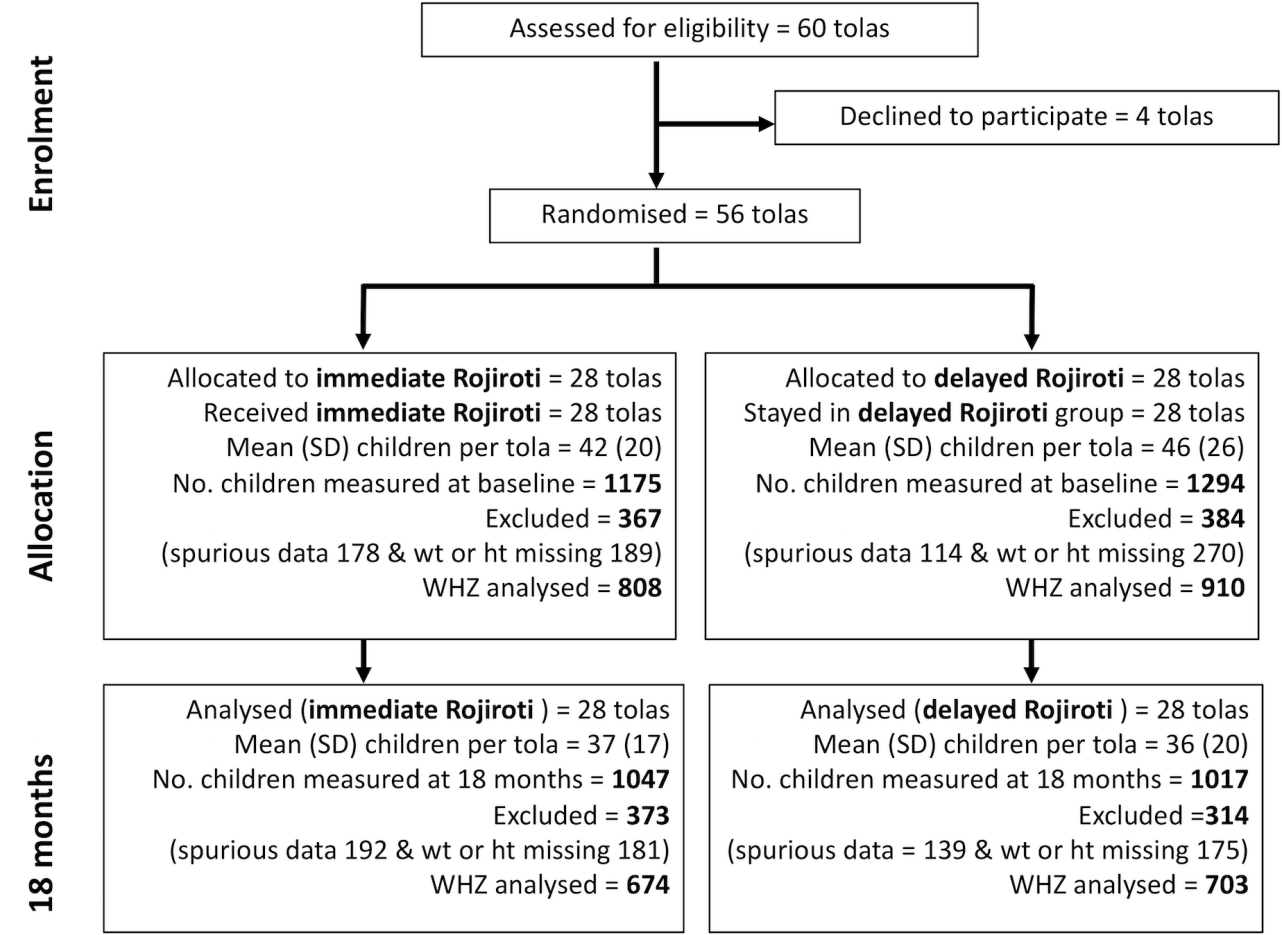
CONSORT diagram for cluster randomised controlled trial of the Rojiroti microfinance programme showing the number of children contributing to the primary outcome (WHZ) at 18 months. WHZ, weight for height Z score.

Between August 2013 and September 2014, 60 tolas were approached, 56 consented and 28 were randomised to each arm (figure 2). All 56 tolas provided data for the primary analysis. The final 18-month follow-up visit was in March 2016. Baseline characteristics of the tolas are in table 1. Baseline data were collected from 2469 children (1560 mothers) in 56 tolas (table 1). Baseline, demographic and nutritional data for the children in the intervention and control arms are shown in table 1. There was no difference between arms in WHZ at baseline (calculated in 1718 of 2469 children), although more children were wasted in the intervention arm (20%) versus controls (15%). Conversely, in the control group, HAZ was worse (−2.14 vs −2.00) and more children had MUAC <12.5 cm (16% vs 13%).

**Table 1:** Baseline characteristics of participating tolas, women and children

Participating tolas	Intervention (n=28)	Control (n=28)	P value
Connected to a paved road (n, %)	28 (100)	27 (96)	0.3
Distance from main road (km) (median, IQR)	1 (0.3–4)	0.9 (0.45–3.5)	0.8
PDS* shop (n, %)	11 (39)	14 (50)	0.4
Government primary school (n, %)	28 (100)	27 (96)	0.3
Other school (n, %)	13 (46)	8 (29)	0.2
Primary health centre (n, %)	0 (0)	0 (0)	–
Presence of an ASHA* worker (n, %)	26 (93)	27 (96)	0.6
Presence of an ANM† (n, %)	26 (93)	25 (89)	0.6
Presence of a water tap or pipeline (n, %)	0 (0)	0 (0)	–
Presence of electricity supply (n, %)	28 (100)	28 (100)	–

Participating women	Intervention (n=756)	Control (n=804)	P value
Number of women per tola (median, IQR)	25.5 (17.5 to 38)	27 (18.5 to 36.5)	0.9
Age in years (mean, SD)	27.6 (5.5)	27.6 (5.3)	0.9
Family owns land (n, %)	100 (13)	64 (8)	0.001
Able to travel outside of tola without permission from male relative (n, %)	61 (8)	27 (3)	<0.001
Able to read and write (n, %)	158 (21)	132 (16)	0.05
Attended school (n, %)	145 (19)	103 (13)	0.001

Participating children	Intervention (n=1175)	Control (n=1294)	P value
Median (IQR) no. of children per tola	43 (27–57)	47 (28–59)	0.6
No. of men, (%)	612 (52)	650 (50)	0.4
Mean (SD) age in months	30.9 (16.8)	31.2 (16.9)	0.6
Born at home (n, %)	356 (30)	359 (28)	0.2
Road to health‡ card (n, %)	1055 (90)	1166 (90)	0.8
Immunised§ (n, %)	1125 (96)	1257 (97)	0.06

Baseline nutritional measures of participating children	Intervention	n Intervention¶	Control	n Control¶	P value**
WHZ (mean, SD)	−1.00 (1.16)	808	−0.94 (1.00)	910	0.3
HAZ (mean, SD)	−2.00 (1.29)	808	−2.14 (1.33)	910	0.03
WAZ (mean, SD)	−1.89 (1.10)	985	−1.96 (1.10)	1170	0.2
MUAC (mean, SD)	13.6 (1.14)	933	13.6 (1.25)	1080	0.5
Wasted (n, %)	159 (20)	808	138 (15)	910	0.01
Stunted (n, %)	399 (49)	808	489 (54)	910	0.07
Underweight (n, %)	436 (44)	985	548 (47)	1170	0.2
MUAC <12.5 cm (n, %)	122 (13)	933	176 (16)	1080	0.04
MUAC <11.5 cm (n, %)	24 (3)	933	44 (4)	1080	0.06

Intervention=immediate Rojiroti. Control=delayed Rojiroti.

*ASHA, Accredited Social Health Activists (local women, trained in health promotion).

†ANM, Auxiliary Nurse Midwife (village level maternal and child health worker).

‡Road to health card is a summary of health and growth of the child in the first 5 years of life.

§Immunisation defined as maternal recall of any immunisation received by the child.

¶The number of children contributing to each outcome measure is given in each row.

**P values from t-test for continuous outcomes and χ^2^ for binary outcomes.

††PDS, public distribution system (a network of subsidised government stores).

HAZ, height for age Z score; MUAC, mid-upper arm circumference; WAZ, weight for age Z score; WHZ, weight for height Z score.

### Cross-sectional analysis of effect of Rojiroti on undernutrition

At 18 months, data were collected from 2064 children under five (1326 mothers) – see figure 2. We excluded 687 children from the analysis of WHZ because their data were either missing or flagged as spurious by the ENA tool; 1377 children were included (anthropometric data shown in table 2).

**Table 2:** Cross-ectional analysis of the effects of the Rojiroti microfinance programme on nutritional status of children under 5 years of age at 18-month follow-up

	Intervention	n	Control	n	Unadjusted* β/OR† (95% CI) Intervention versus control at follow-up	P value	ICC‡	Intervention n	Control n	Adjusted* β/OR† (95% CI) Intervention versus control at follow-up	P value	ICC‡
WHZ (mean, SD)	−1.02 (1.11)	674	−1.37 (1.10)	703	β=0.38 (0.16 to 0.61)	0.001	0.108	259	300	β=0.25 (-0.03 to 0.53)	0.08	0.175
HAZ (mean, SD)	−2.37 (1.29)	674	−2.53 (1.25)	703	β=0.17 (−0.04 to 0.37)	0.1	0.053	259	300	β=−0.07 (−0.24 to 0.10)	0.4	0.068
WAZ (mean, SD)	−2.13 (1.03)	842	−2.37 (1.05)	871	β=0.27 (0.11 to 0.43)	0.001	0.051	356	433	β=0.26 (0.04 to 0.49)	0.02	0.212
MUAC (mean, SD)	13.6 (1.10)	811	13.4 (1.12)	828	β=0.22 (0.03 to 0.40)	0.02	0.063	331	379	β=0.12 (−0.14 to 0.38)	0.4	0.279
Wasted (n, %)	122 (18)	674	200 (29)	703	OR=0.46 (0.28 to 0.74)	0.002	0.134	259	300	OR=0.61 (0.33 to 1.14)	0.1	0.135
Stunted (n, %)	421 (63)	674	465 (66)	703	OR=0.82 (0.60 to 1.12)	0.2	0.044	259	300	OR=0.97 (0.57 to 1.64)	0.9	0.056
Underweight (n, %)	446 (53)	842	545 (63)	871	OR=0.63 (0.47 to 0.84)	0.002	0.042	356	433	OR=0.51 (0.29 to 0.89)	0.02	0.155
MUAC <12.5 cm (n, %)	102 (13)	811	152 (18)	828	OR=0.65 (0.41 to 1.05)	0.08	0.116	331	379	OR=0.78 (0.27 to 2.23)	0.6	0.320
MUAC <11.5 cm (n, %)	24 (3)	811	37 (5)	828	OR=0.70 (0.36 to 1.33)	0.3	0.097	331	379	OR=0.79 (0.10 to 6.14)	0.8	0.289

Not all outcome measures could be recorded for each child and so the number of children contributing to each outcome is listed.

Supine length or standing height was measured in 81% of children at baseline and 79% at follow-up. Weight was measured in 99% of children at baseline and 99% at follow-up. In children aged 6–59 months, MUAC was measured in 99% of children at baseline and 99% at follow-up.

Control=delayed Rojiroti.

Intervention=immediate Rojiroti.

*Adjusted for baseline values of the outcome, age, sex and number of children under 5 in family.

†β is the regression coefficient for continuous outcomes, and OR is the odds ratio, both adjusted for clustering.

‡ICC is the intracluster correlation coefficient (proportion of the total variance of the outcome that can be explained by the variation between clusters).

HAZ, height for age Z score; MUAC, mid-upper arm circumference; WAZ, weight for age Z score; WHZ, weight for height Z score.

In the unadjusted analysis, the primary outcome (mean WHZ in children under five) at 18 months was significantly higher in the intervention than control tolas (−1.02 vs −1.37, regression coefficient, adjusted for clustering β=0.38, 95% CI 0.16 to 0.61, p=0.001). The WHZ in the control arm deteriorated, compared with baseline, while the intervention arm showed little change. In the unadjusted analysis, the following secondary outcomes were also significantly better in the intervention tolas compared with controls (table 2): mean WAZ (−2.13 vs −2.37), MUAC (13.6 cm vs 13.4 cm), prevalence of wasting (18% vs 29%) and underweight (53% vs 63%).

Table 2 also shows the analysis adjusted for baseline nutritional measures. In this analysis, there were significant differences only in favour of the intervention for WAZ (β=0.26, 95% CI 0.04 to 0.49, p=0.02) and the prevalence of underweight (OR=0.51, 95% CI 0.29 to 0.89, p=0.02). Nutritional outcomes were similar in children in intervention tolas whether their mothers were members of a Rojiroti SHG. There were no differences with non-Rojiroti SHG membership (online supplementary table).

10.1136/archdischild-2018-316471.supp2 Supplementary data

### Outcomes for women

**Figure 3:**
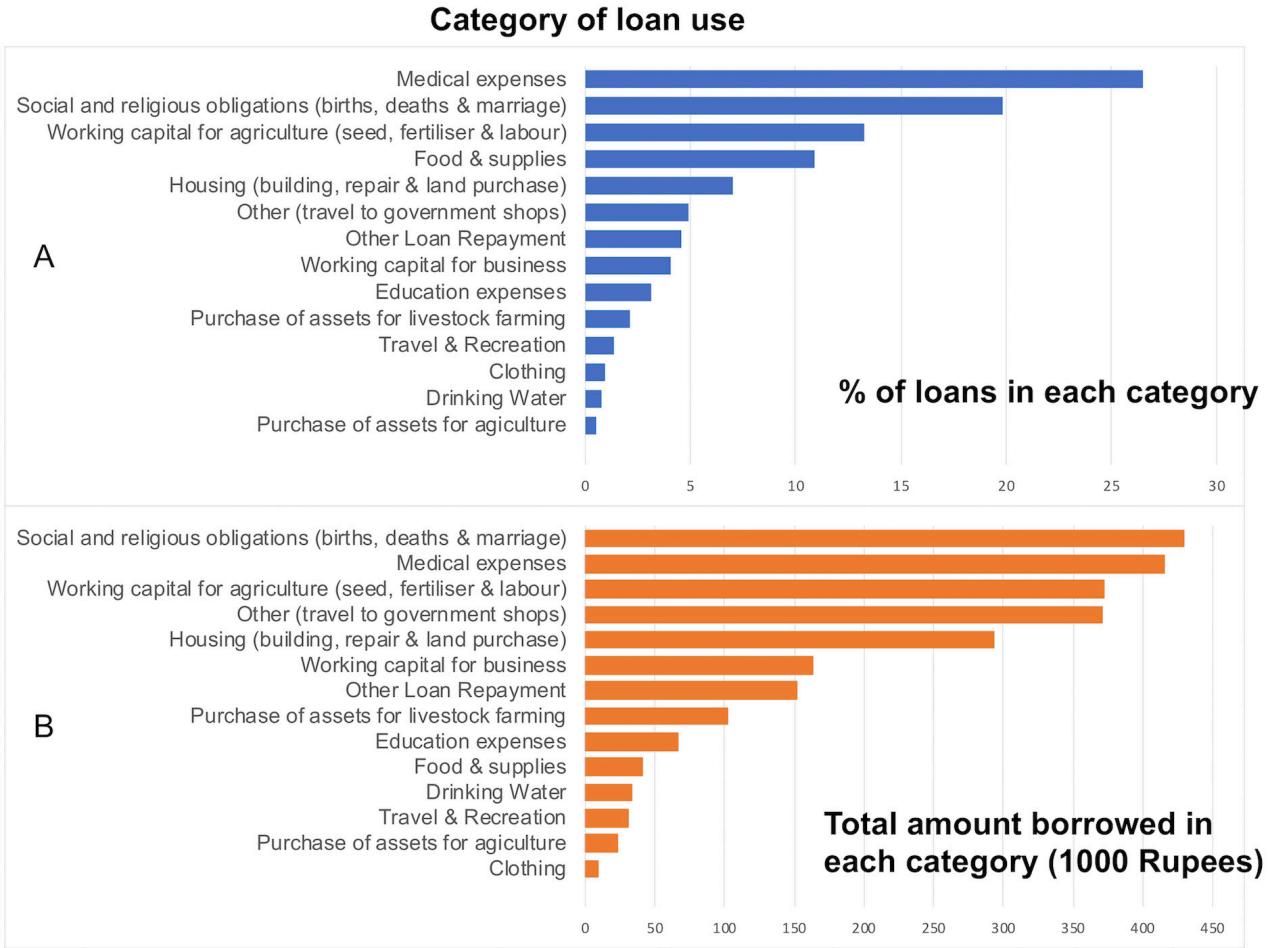
Use of loans by women in the intervention tolas, showing % of loans granted (A) and total amount borrowed in each category (B).

At 18 months, 33 (5%) women in the intervention group and 31 (5%) in the control group were free to travel without the permission of a male relative (p=0.5). Forced asset sales during the study period were similar in the two groups: intervention: 16 women (2%) versus control 15 (2%) women (p=0.8). There were 1134 loans, and the total borrowed was Rs2 499 532 ($36 858). The mean loan value was Rs2 204 ($32). figure 3 shows the percentage of loans (A) and the amount borrowed (B) for each category of use.

## Discussion

Our cluster randomised trial showed better child nutrition in tolas that received Rojiroti microfinance. In an unadjusted analysis, there was a difference of 0.35 in mean WHZ, and only 18% of children in the intervention group were wasted versus 29% of controls. After adjusting for baseline nutrition, in the subgroup with data at baseline and follow-up, differences in were seen for WAZ and the prevalence of underweight only. Fewer children had both baseline and follow-up data available for the adjusted WHZ analysis (n=559) compared with those with data available for follow-up only (n=1377). This was also true in the analyses of other outcome measures. This is likely to be due to a high proportion of the population of rural Bihar moving repeatedly to find work, with families frequently moving in or out of each tola.

A recent, systematic review of the health impacts of group-based microfinance programmes found no randomised trial, with child health outcomes.^18^ There has been little rigorous, evaluation of the impact of microfinance.^19 20^ Our study is the first RCT to evaluate the effects of microfinance on child nutrition. The only previous randomised study^21^ did not consider child health outcomes. Of the non-randomised studies, with child health outcomes, some have suggested a benefit,^22–24^ while others have shown either no benefit^25^ or worse nutrition^26^ in the children of microfinance participants.

Nutritional indices did not improve in the intervention group but worsened in controls, suggesting that Rojiroti prevented deterioration in nutritional status. For phase 1 and three tolas, the 18-month follow-up occurred just before the Rabi harvest (wheat and lentils, March–April).^27^ For phase 2 tolas, the final visit was 2 months before the Kharif harvest (rice and lentils, November–December).^27^ The scarcity of staple foods at follow-up may explain the deterioration in the control group. Similar seasonal variations in children’s growth have been observed in rural Africa.^28^ Rojiroti microfinance may confer resilience on the community during periods of shortage.

This trial has limitations. The results may not be applicable outside Bihar. Our primary outcome was mean WHZ in the intervention and control tolas at 18 months. Many children measured at 18 months had not been present at baseline and so our analysis adjusting for baseline nutrition was conducted in a much smaller group. Also, we do not know what proportion of the children in each village attended for weighing and measuring. We included data from phase 1 (feasibility) in our final analysis, as the power calculation, based on phase 1 data, indicated this trial could reach a definitive conclusion. Data on length or height were missing in around 20% of children (mostly too young to stand). At baseline, we excluded 751 of 2469 children, and at 18 months 687 of 2064 children, from WHZ analysis because of spurious or missing data (figure 2). In a future trial, we will have increased quality assurance to reduce errors in measurement and recording. Rather than applying a correction for multiple hypothesis testing, we have presented full data, with 95% CIs and p values, to allow the reader to judge the weight of evidence. The nutritional benefit in children was seen whether their mothers received the intervention. This may be attributable to a ‘trickle down’ of wealth in the community but might also be due to the higher degree of landownership, literacy and schooling that occurred, by chance, in the women comprising the intervention group.

The defining characteristics of Rojiroti must be understood, if the approach is to be used elsewhere (see box 1). The small sums and time-consuming meetings mean that Rojiroti is attractive only to the very poor. There are no restrictions on the use of loans and yet repayment rates are around 99%.^8^ The price of defaulting on a loan is not loss of the borrower’s property but loss of access to affordable credit. Rojiroti is therefore distinct from women’s groups linked to cash transfer.^29^

Women only agreed to participate because they knew that tolas allocated to control would receive Rojiroti after 18 months. We do not know if benefits are seen beyond the 18 months. We have shown the percentage of loans and amounts borrowed in each category (figure 3). By both criteria, the three most common uses are medical expenses, social and religious obligations and working capital for agriculture. Future research should evaluate effects on harms (such as domestic violence) and how access to credit for some mothers can benefit the children in the whole community. Our theory of change diagram (online supplementary figure) shows how these factors may interact and postulates mechanisms for the Rojiroti effect.

Women participants were very poor (10% land ownership), and there were very low levels of decision making agency (only 5% could travel without permission). Less than 20% were literate versus 46% literacy among most rural women in Bihar.^12^ Children showed a higher prevalence of wasting, stunting, underweight and moderate malnutrition than reported in the National Family Health Survey 4.^12^ The Rojiroti approach is designed for very poor communities and may not show the same benefits where poverty is less extreme.

Rojiroti has grown organically in Northern India over the last 15 years. There are now approximately 31 000 members in 3100 SHGs in Bihar.^8^ Scaling up the intervention can happen with modest funding and could deliver better health for children.
