# Supplementary material for: Rojiroti microfinance and child nutrition: a cluster randomised trial
Source: Arch Dis Child. 2019 Oct 10;105(3):229–35. doi: 10.1136/archdischild-2018-316471 (PMC7041497; doi:10.1136/archdischild-2018-316471)
Supplement: Supplementary data [file archdischild-2018-316471supp001.pdf]

SUPPLEMENTARY STATISTICAL ANALYSIS

Rojiroti microfinance & child nutrition: a cluster randomised trial.

Authors: Shalini Ojha, Lisa Szatkowski, Ranjeet Sinha, Gil Yaron, Andrew Fogarty, Stephen Allen, Sunil Choudhary, and Alan R Smyth

SUPPLEMENTARY METHODS

Effects of mothers' SHG membership on nutritional outcomes in children under 5 years.

In order to examine whether any effects of the intervention varied according to mothers' SHG membership, linear and logistic regression analysis were repeated comparing all children measured at baseline in intervention and control tolas using a four level exposure variable:

- control and mother not a member of an SHG (reference category)
- control but mother a member of a non-Rojiroti SHG
- mother in intervention tola and member of a Rojiroti SHG
- mother in intervention tola but not a member of a Rojiroti SHG

SUPPLEMENTARY RESULTS

Effects of mothers' SHG membership

In the intervention group, 35% of women overall (median by tola 37%, interquartile range 8% - 59%) reported being members of a Rojiroti SHG. In control tolas, 29% of women overall (median by tola 24%, interquartile range 0% - 54%) reported being a member of a non-Rojiroti SHG. Comparisons of the mean z-scores and odds ratios for binary outcomes at follow-up in children in intervention and control tolas according to maternal SHG membership are given in the Supplementary Table. The regression coefficients and odds ratios were similar in children in intervention tolas in both the children of women who were members of a Rojiroti SHG and the children of women who were not members. There were no significant differences between children of women in control tolas who were members of a non-Rojiroti SHG and those who were not.

	Mean z-score (β, 95% CI for group vs. control)				p-value difference between groups
	**Control non-SHG member**	**Control SHG member**	**Intervention non-SHG member**	**Intervention SHG member**	
**WHZ**	-1·35 (ref)	-1·45 (-0·10, -0·31 to 0·11)	-1·02 (0·36, 0·11 to 0·61)	-0·99 (0·32, 0·06 to 0·59)	0·01
**HAZ**	-2·53 (ref)	-2·55 (0·00, -0·23 to 0·24)	-2·37 (0·15, -0·09 to 0·39)	-2·35 (0·21, -0·05 to 0·47)	0·4
**WAZ**	-2·35 (ref)	-2·44 (-0·08, -0·26 to 0·09)	-2·16 (0·22, 0·04 to 0·40)	-2·05 (0·29, 0·09 to 0·49)	0·007
**MUAC (cm)**	13·4 (ref)	13·4 (-0·03, -0·22 to 0·16)	13·6 (0·15, -0·06 to 0·36)	13·7 (0·25, 0·02 to 0·47)	0·1
	**% (OR, 95% CI for group vs. control)**				**p-value difference between groups**
**Wasted**	26 (ref)	33·5 (1·42, 0·91 to 2·22)	17·2 (0·48, 0·28 to 0·84)	19·1 (0·60, 0·33 to 1·09)	0·007
**Stunted**	66 (ref)	68·0 (1·05, 0·71 to 1·57)	63·4 (0·89, 0·62 to 1·28)	60·3 (0·73, 0·49 to 1·09)	0·4
**Underweight**	63 (ref)	62·9 (1·02, 0·72 to 1·45)	55·8 (0·71, 0·51 to 0·99)	46·6 (0·52, 0·36 to 0·75)	0·003
**MUAC <12.5cm**	17 (ref)	19·2 (1·18, 0·74 to 1·87)	13·9 (0·77, 0·45 to 1·30)	10·2 (0·65, 0·35 to 1·20)	0·4
**MUAC <11.5cm**	5 (ref)	3·4 (0·73, 0·31 to 1·74)	3·4 (0·78, 0·37 to 1·66)	2·2 (0·55, 0·20 to 1·51)	0.7

CI, confidence interval; OR, odds ratio; ref. reference group; WHZ, weight for height z-score; HAZ, height for age z-score; WAZ, weight for age z-score; MUAC, mid-upper arm circumference; wasted, WHZ <-2SD; stunted, HAZ<-2SD; underweight, WAZ<-2SD

Supplementary Table. *Comparison of mean z-scores and odds ratios at follow-up in children in intervention vs. control tolas according to mother's self-help group membership.*

Supplementary Figure. Theory of change diagram of how Rojiroti may improve child nutrition. Intermediate & longer term outcomes are "assumptions" apart from those shaded which have been demonstrated in our field study.[1]

REFERENCES

1. Yaron G, Gordon R, Best J, Choudhary S. Microfinance for the marginalized: the impact of the Rojiroti approach in India. *Enterprise Development and Microfinance.* 2018;29(1):80-93. https://www.developmentbookshelf.com/doi/abs/10.3362/1755-1986.17-00011
