# Supplementary figures and images for: Rojiroti microfinance and child nutrition: a cluster randomised trial
Source: Arch Dis Child. 2019 Oct 10;105(3):229–35. doi: 10.1136/archdischild-2018-316471 (PMC7041497; doi:10.1136/archdischild-2018-316471)

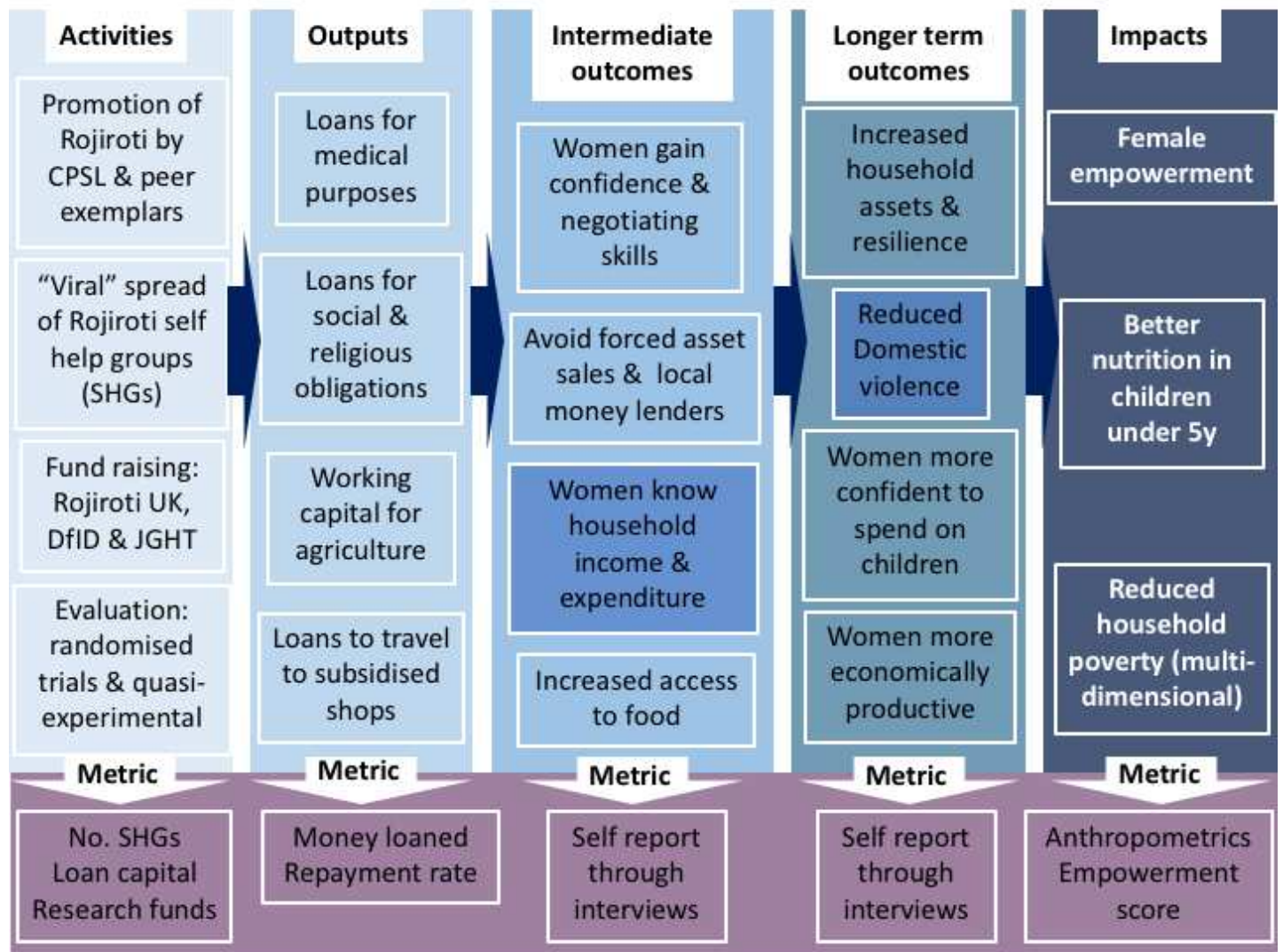

Supplement: Supplementary data [file archdischild-2018-316471supp002.pdf]
